# Spatial variation in fertilizer prices in Sub-Saharan Africa

**DOI:** 10.1371/journal.pone.0227764

**Published:** 2020-01-14

**Authors:** Camila Bonilla Cedrez, Jordan Chamberlin, Zhe Guo, Robert J. Hijmans

**Affiliations:** 1 Department of Environmental Science and Policy, University of California, Davis, California, United States of America; 2 International Maize and Wheat Improvement Center (CIMMYT), Nairobi, Kenya; 3 International Food Policy Research Institute (IFPRI), Washington, DC, United States of America; United Nations University Institute for Natural Resources in Africa, GHANA

## Abstract

Low crop yields in Sub-Saharan Africa are associated with low fertilizer use. To better understand patterns of, and opportunities for, fertilizer use, location specific fertilizer price data may be relevant. We compiled local market price data for urea fertilizer, a source of inorganic nitrogen, in 1729 locations in eighteen countries in two regions (West and East Africa) from 2010–2018 to understand patterns in the spatial variation in fertilizer prices. The average national price was lowest in Ghana (0.80 USD kg^-1^), Kenya (0.97 USD kg^-1^), and Nigeria (0.99 USD kg^-1^). Urea was most expensive in three landlocked countries (Burundi: 1.51, Uganda: 1.49, and Burkina Faso: 1.49 USD kg^-1^). Our study uncovers considerable spatial variation in fertilizer prices within African countries. We show that in many countries this variation can be predicted for unsampled locations by fitting models of prices as a function of longitude, latitude, and additional predictor variables that capture aspects of market access, demand and environmental conditions. Predicted within-country urea price variation (as a fraction of the median price) was particularly high in Kenya (0.77–1.12), Nigeria (0.83–1.34), Senegal (0.73–1.40), Tanzania (0.90–1.29) and Uganda (0.93–1.30), but much lower in Burkina Faso (0.96–1.04), Burundi (0.95–1.05), and Togo (0.94–1.05). The correlation coefficient of the country level models was between 0.17 to 0.83 (mean 0.52) and the RMSE varies from 0.005 to 0.188 (mean 0.095). In 10 countries, predictions were at least 25% better than a null-model that assumes no spatial variation. Our work indicates new opportunities for incorporating spatial variation in prices into efforts to understand the profitability of agricultural technologies across rural areas in Sub-Saharan Africa.

## Introduction

Crop yields in Sub-Saharan Africa (SSA) are generally very low [1, 2]. For example, the average cereal yield in SSA is 1266 kg ha^-1^ while the global average is 3745 kg ha^-1^ [3]. There are several factors contributing to this, but low soil fertility stands out and the increased use of inorganic fertilizers could strongly increase crop yields [4, 5, 6, 7]. The average application rate of inorganic fertilizer on arable land in Sub-Saharan Africa is 14 kg ha^-1^, much lower than the 141 kg ha^-1^ in South Asia, 154 kg ha^-1^ in the European Union, 175 kg ha^-1^ in South America, and 302 kg ha^-1^ in East Asia [3].

A number of hypotheses have been proposed to explain the low usage of inorganic fertilizer in SSA: (a) on many African soils, crops do not respond well to fertilizer, perhaps because of a low soil organic matter content or soil degradation [8, 9, 10, 11, 12]; (b) farmers are not aware of, or do not believe in the utility of inorganic fertilizer [13, 14, 15]; (c) farmers have a cash-flow problem, and need better access to credit to buy fertilizers [13, 16, 17]; (d) variability in rainfall makes it too risky to invest in fertilizers, and insurance programs may be needed to support fertilizer use [5, 18]; and (e) after accounting for agronomic responses to fertilizer; local input and output prices are such that fertilizer investments are insufficiently profitable for many farmers [18, 19, 20, 21].

It is challenging to evaluate these hypotheses, because each one of them may be true in some location or under certain conditions, and because the location specific data needed for analysis is not readily available. For example, to evaluate the economic returns to fertilizer in a particular location, we need to understand crop responses to fertilizer under local conditions, as well as the effective price of fertilizer, and the price that a farmer could get for the crop products.

In this paper we focus on one particular data gap: the spatial variation in the price of fertilizer. While national level fertilizer prices may be available, we need to consider the extent to which prices vary within countries, reflecting transportation costs and other factors [22, 23, 24, 25]. In the absence of such data, simplifying assumptions are made that may not be valid. For example, evaluations of the returns to production technologies in developing country settings have often assumed spatially invariant input and output prices (e.g., [26, 27]) or modeled spatial variation in prices based on assumptions about price variation as a function of distance to markets (e.g. [24, 28, 29]).

An obstacle to using empirical data on sub-national variation in fertilizer prices is the scarcity of such data. A few studies have utilized price variation obtained from averaging price survey data over arbitrary sub-national boundaries, such as districts or regions (e.g. [18, 30, 31]), but, to our knowledge, there has not been an attempt to systematically describe the variability of fertilizer prices within countries and our ability to estimate the price at unsampled locations.

To address this gap, we compiled local fertilizer price data from eighteen countries in SSA. The main goal of this paper was to describe price variation within and between countries and to use spatial interpolation models to predict local prices of urea, a commonly used source of inorganic nitrogen. In addition to the main goal of investigating whether we can interpolate price data, we analyzed the relationship between urea and other fertilizer type and between subsidized and non-subsidized fertilizer prices.

Our models enable us to predict prices at locations for which there are no empirical data available. The amount of sub-national price variation, and the performance of our interpolation models, differs strongly across countries in our sample. In countries where there is consistent regional price variation, we show that interpolation methods can be used to estimating unobserved local prices. The spatial price predictions–“price maps”–that are generated by our approach can be used to improve empirical research on technology use (by more accurately approximating local input and output prices faced by farmers), as well as incorporated into targeting and planning frameworks for agricultural investments (e.g. by targeting promoting efforts to areas where technologies are most profitable).

## Materials and methods

### Price data

We compiled fertilizer price data for eighteen countries in West and East Africa from two data sources: (1) Africa Fertilizer [32] and (2) the Living Standards Measurement Study-Integrated Surveys on Agriculture (henceforth LSMS) [33].

Africa Fertilizer reports prices over time for major towns in different countries. Prices are for 25 kg or 50 kg bags and expressed in the national currency. We compiled 7823 observations for 878 locations in seventeen countries: Benin, Burundi, Burkina Faso, Côte d’Ivoire, Ghana, Kenya, Mali, Malawi, Mozambique, Niger, Nigeria, Rwanda, Senegal, Togo, Tanzania, Uganda, and Zambia from 2010–2018. We used the town name to assign geographic coordinates to each location. Africa Fertilizer reported non-subsidized and subsidized prices for several countries.

LSMS-ISA (hereinafter “LSMS”) is a nationally representative multi-topic household survey implemented in eight countries in SSA: Burkina Faso, Ethiopia, Malawi, Mali, Niger, Nigeria, Tanzania, Uganda [33]. For Ethiopia, Mali, and Malawi, prices were reported as the total amount paid for a quantity of fertilizer purchased expressed in the national currency. We used that information to calculate the price of fertilizer per kg. For Nigeria and Tanzania, the questions about the purchased fertilizer (i.e. type, source, and amount used) were at the plot level, while the reported price is for the total quantity of purchased fertilizer. To compute the fertilizer price, we assumed that the amount of fertilizer used on all plots was equal to the total amount purchased by the farmers. We did not use the LSMS data for Burkina Faso and Uganda, as the type of inorganic fertilizer purchased was not specified. We did not use the data for Niger as the locations of the households were not provided, and there were only 13 households that reported a price for urea, which is our main focus in this paper.

Different types of fertilizer are reported by LSMS and Africa Fertilizer depending on the country, e.g. urea, calcium ammonium nitrate, diammonium phosphate, and ‘NPK’ (in the case of LSMS without specifying the N, P or K content). For this study, we focus on urea price because it is the product with most observations across all the countries studied and because it is an unambiguous product (unlike, for example, fertilizers labeled as “NPK” which may vary widely in composition percentages). Urea [CO(NH_2_)_2_] is the world’s most widely used form of inorganic nitrogen fertilizer. It contains 46% N, and no other plant nutrients. We compared the price of urea with that of “NPK” by including all the ‘NPK’ formulations reported by Africa Fertilizer, with CAN (calcium ammonium nitrate) and DAP (diammonium phosphate). CAN is also known as nitro-limestone. The chemical formula is variable (e.g. [NH_4_NO_3_ + CaCO_3_ * MgCO_3_]) but in general it contains 8% of Ca and 21–27% of N as [NO_3_^-^] and [NH_4_^+^]. DAP [(NH_4_)_2_HPO_4_] is the world’s most widely used phosphorous fertilizer and is also a source of N. It contains 18% N and 20% P.

### Data manipulation

To allow for comparison of prices between years and countries, we converted prices from national currency to inflation-adjusted purchasing power parity United States dollars (USD) by dividing them by the consumer price index (CPI) times the purchasing power parity (PPP) dollar value for each year according to the World Bank [3]. The CPI is defined as the changes in the cost to the average consumer of acquiring a basket of goods and services that may be fixed or changed between years. Our base year was 2010 (CPI = 1). A weakness of the CPI is that it may not be a very accurate measure of price changes in more rural areas. The PPP conversion factor represents how much of a country’s currency (expressed in USD using the international exchange rate) is required to buy the same amounts of goods and services in the domestic market as one USD would buy in the United States.

To improve the quality of the data, we removed gross outliers that were clearly implausible. Outliers were defined as values that were more than 1.5 times the distance between the lower and upper quartiles away from the lower and upper quartile.

### Comparison of fertilizer prices

Although we focus on the price for urea, we provide some comparison with the price for other fertilizers. For the Africa Fertilizer data set, we fitted: (a) a liner regression model with no intercept between the prices for urea and the other fertilizer types, for locations where both the urea price and that of another type were reported; (b) the same linear regression models for each country separately; and (c) a liner regression model with no intercept between subsidized and non-subsidized urea prices, for each country separately. We use no intercept because that gives a simple number that can be readily interpreted (the price of y is a*x). Also, the implicit (0, 0) intercept should be correct for prices.

### Spatial prediction

To study spatial variation in prices, we first removed temporal variation by calculating a spatial price index computed as the price in a location divided by the national median price for that year. For spatial predictions we focused on the non-subsidized urea prices for 2014 to 2018. We build predictive models of the spatial price index using location (longitude and latitude) and additional predictor variables that capture aspects of market access (travel time to market and distance to port), demand for product (population density and cropland) and one of the environmental factors that influence crop responses to fertilizer (precipitation). All predictor variables were organized as spatial raster data sets with a 5 arc-minute (about 9 × 9 km) spatial resolution.

Specific market access variables used were travel time in hours to (1) the nearest port, (2) the nearest town with over 50,000 inhabitants, (3) the nearest town with over 100,000 inhabitants and (4) over 250,000 inhabitants [34]. Population and rural population density estimates were measured in persons per km^2^ [35]. Cropland was computed by aggregating a 30 m spatial resolution crop mask data [36] so that the values represent the fraction of land that is used for crop production and smoothed by computing a (5x5 cells) focal average. We used annual precipitation (mm) from WorldClim version 2 [37]. The spatial data was handled with the “raster” package [38]. For the regional models we also used median country level price as a predictor variable.

For each country and region (West and East Africa), Random Forest Regression and Thin Spline Plate algorithms were used to fit models. Random Forest has the benefit of flexibility for fitting potentially irregular surfaces resulting from complex interactions [39]. We used the Random Forest algorithm as implemented in the R-package ‘randomForest’ [40]. The tuneRF function was used to find the optimal number of variables available for splitting at each tree node (the ‘mtry’ parameter). Random Forest models tend to predict very well within the range of the observed values, but the predictions may show sudden breaks, and can be poor in extrapolating. Therefore, we also used Thin Plate Splines (TPS) to create smooth surfaces. TPS is a flexible local regression method that lends itself to interpolating noisy data with high levels of uncertainty [41]. The model is fit by minimizing the residual sum of squares subject to a constraint that the function has a certain level of smoothness (to avoid overfitting). The required level of smoothness is set by the roughness parameter which is determined through cross-validation. This, while producing smooth surfaces, TPS will not necessarily return the observed value for a sampled point. This helps guard against over-fitting especially for noisy datasets where the observations themselves are estimates based on small samples [42]. The TPS model was implemented using the ‘Tps’ function in the R-package ‘fields’ [43] with only longitude and latitude as predictor variables. We combined the Random Forest and TPS models into a single ensemble model using the weighted average of the inverse Root Mean Square Error (RMSE) of each model. We predicted the spatial price index and derived absolute prices from that by multiplying it with the current average price.

We evaluated the models with five-fold cross-validation. We computed Pearson’s correlation coefficient and Root Mean Square Errors (RMSE) as test statistics. We compared the RMSE of the interpolation (E_I_) with that of a Null-model (E_0_), in which we assumed no spatial price variation, by computing relative RMSE as E_R =_ 1-(E_I_ / E_0_). E_R_ expresses how much better the interpolated predictions are relative to the using a single national price. We build separate models for the Africa Fertilizer and LSMS data. For Tanzania, we also fitted a third model by combining Africa Fertilizer and LSMS data, used a sample from LSMS with the sample size equal to number of observations in the Africa Fertilizer data for Tanzania. For the regional model, there were many more samples in the countries for which we had LSMS data. To avoid an imbalance in the model evaluation, we used a sub-sample of the test data for these countries, with the sample size equal to the mean number of observations for the non-LSMS countries in the region.

Variable importance was assessed for the Random Forest model using the percentage increase in Mean Squared Error, that is, the increase in MSE if the variable had no information.

### Transportation costs

For Ethiopia, Nigeria, and Tanzania, LSMS reported where the farmers purchased the fertilizer (i.e. within or near the village, within or near the nearest urban center, outside the district or region). In addition, for Nigeria and Malawi LSMS reported transportation used by farmers to get to the market and transportation cost. We calculate the proportion of different transportation type used by farmers and computed average transportation cost of each transportation type. We fitted a linear regression model with no intercept between transportation cost and travel time (in hours) to market.

## Results

### Data quality

We retrieved 12659 observations of farmer-reported urea prices from LSMS and retained 10528 observations. The LSMS data for Mali and Nigeria had many more outliers (25% and 24%) than Ethiopia (13%), Tanzania (10%), and Malawi (1.6%) (Table 1). Most outliers were extreme prices: below 0.1 USD kg^-1^ or above 5 USD kg^-1^. In Nigeria, 48% of the outliers were associated with small quantities (10 kg or less) and 35% were associated with quantities below 4 kg. Even though prices could be expected to be higher per unit weight for low quantities purchased, they remained excessive. In some cases, there appeared to have been a mix up in the units between kg and several reports of purchases of 0.5, 1, 2, 3 or 4 kg of fertilizer, probably referred to the number of 50 kg bags instead. For example, a purchase of 2 kg of urea for 79 USD is unlikely, whereas dividing that price by 100 kg (for two bags of 50 kg) yields a plausible price of 0.79 USD kg^-1^. However, many observations associated with these amounts cannot be fixed by multiplying with 50, because even after changing the units to number of bags, the prices remained too high. In addition, there were other cases where low amounts of fertilizer were associated with low prices (less than 0.1 USD kg^-1^).

**Table 1:** Number of observations collected for urea prices, number of observations of non-subsidized urea, number of observations after removing outliers (*n*) and number of observations after aggregating by household (values between parenthesis), and the percent of outliers detected (%) for each data source.

Country	Observations	Non-subsidized	*n*	Outliers (%)	Data source
Benin	53	43	41	5	Africa Fertilizer
Burkina Faso	132	90	90	0	Africa Fertilizer
Burundi	137	87	66	24	Africa Fertilizer
Côte d’Ivoire	80	80	80	0	Africa Fertilizer
Ethiopia	7768	7768	6788 (2045)	13	LSMS
Ghana	150	85	83	2	Africa Fertilizer
Kenya	123	123	122	1	Africa Fertilizer
Malawi	142	119	113	5	Africa Fertilizer
Malawi	1071	1071	1054 (1047)	1.6	LSMS
Mali	122	77	77	0	Africa Fertilizer
Mali	800	800	604 (309)	25	LSMS
Mozambique	103	103	96	7	Africa Fertilizer
Niger	27	27	27	0	Africa Fertilizer
Nigeria	115	109	109	0	Africa Fertilizer
Nigeria	1864	1864	1418 (1135)	24	LSMS
Rwanda	223	88	88	0	Africa Fertilizer
Senegal	97	81	74	9	Africa Fertilizer
Tanzania	157	143	137	4	Africa Fertilizer
Tanzania	1156	736	664 (515)	10	LSMS
Togo	45	26	26	0	Africa Fertilizer
Uganda	121	121	121	0	Africa Fertilizer
Zambia	194	109	83	24	Africa Fertilizer
**Total**	**14680**	**13750**	**6484**		

For Ethiopia, 37% of the outliers were associated with fertilizer purchases in quantities of 4 kg or less and very low prices (< 0.1 USD kg^-1^). Other low prices (14%) were often associated with large quantities (> 2000 kg) of purchased fertilizer that would seem unlikely for most farmers. The outliers were not clustered in a specific region of the country.

The data from Africa Fertilizer corresponding to non-subsidized urea did not have many outliers, except for Burundi and Zambia (24%); we retained 1433 records of this data source (Table 1).

### Country level prices

#### Urea price

We had most observations on non-subsidized prices for Ethiopia (2045), Nigeria (1135), Malawi (1142), Tanzania (515), and Mali (386) due to the large sample of fertilizer users in the LSMS data for these countries (in addition to the Africa Fertilizer data available for two of these countries). For most other countries, we had between 60 and 137 observations. The lowest number of observations was for Benin (41), Niger (27), and Togo (26) (Table 2). The non-subsidized mean urea price was lower in West Africa than in East Africa (1.07 ± 0.29 vs. 1.24 ± 0.35 USD kg^-1^; t-test *P*<0.05). Ghana, Kenya, and Nigeria had the lowest national mean prices (0.80, 0.97, 0.99 USD kg^-1^). The three countries with the most expensive urea are landlocked (Burundi: 1.51, Uganda: 1.49 and Burkina Faso: 1.49 USD kg^-1^) (Table 2).

**Table 2:** Number of observations, median, mean price, and coefficient of variation (CV) for non-subsidized urea price (USD kg^-1^) per country. Subsidized urea price (median-USD kg^-1^), percentage of the non-subsidized urea price, and number of observations per country (values between parenthesis). Slope (USD kg^-1^) of the regression models between subsidized and non-subsidized prices.

Country	n	Median	Mean	CV	Subsidized price (%), (n)	Slope
Benin	41	1.12	1.17	20	0.80 (71), (10)	0.78
Burkina Faso	90	1.48	1.49	12	1.09 (74), (42)	0.75
Burundi	66	1.52	1.51	20	0.96 (63), (50)	0.61
Côte d’Ivoire	80	1.16	1.23	16	---	---
Ethiopia	2045	1.25	1.23	16	---	---
Ghana	83	0.80	0.80	29	0.56 (70), (65)	0.72
Kenya	122	0.84	0.97	38	---	---
Malawi	1142	1.31	1.19	41	0.12 (9), (23)	0.18
Mali	386	1.02	1.08	18	0.94 (70), (45)	0.69
Mozambique	96	1.36	1.39	22	---	---
Niger	27	1.34	1.35	7	---	---
Nigeria	1135	1.00	0.99	26	0.43 (53), (6)	0.61
Rwanda	88	1.26	1.32	14	1.06 (84), (135)	0.77
Senegal	74	1.38	1.38	25	0.70 (51), (16)	0.62
Tanzania	515	1.36	1.35	30	0.81 (60), (14)	0.68
Togo	26	1.48	1.41	23	0.84 (57), (19)	0.62
Uganda	121	1.42	1.49	30	---	---
Zambia	83	1.32	1.32	28	0.24 (18), (85)	0.23

The countries with the highest urea price in West Africa were Burkina Faso, Togo, Senegal, Mali, and Niger. Except for Senegal, these countries are neighbors and use a common currency (West African CFA franc). Ghana is also a neighbor of three of these countries, but it had the lowest urea prices in West Africa. In East Africa, the countries with the highest prices were Burundi and Uganda while Kenya had the lowest average price in East Africa.

The coefficient of variation (CV) in prices was above 25% for Ghana, Kenya, Malawi, Nigeria, Senegal, Tanzania, Uganda, and Zambia. The CV was relatively low in Burkina Faso, Côte d’Ivoire, Ethiopia, Mali, Niger, and Rwanda (Table 2).

From 2010 to 2018, countries in which Africa Fertilizer reported subsidized prices were: Benin, Burkina Faso, Burundi, Ghana, Malawi, Mali, Nigeria, Rwanda, Senegal, Tanzania, Togo, and Zambia (Table 2). Subsidized prices were much lower than non-subsidized prices in Malawi (9%) and Zambia (18% of the non-subsidized price). In contrast, they were 70% in Ghana and Mali, 71% in Benin, and 84% in Rwanda.

**Fig 1:**
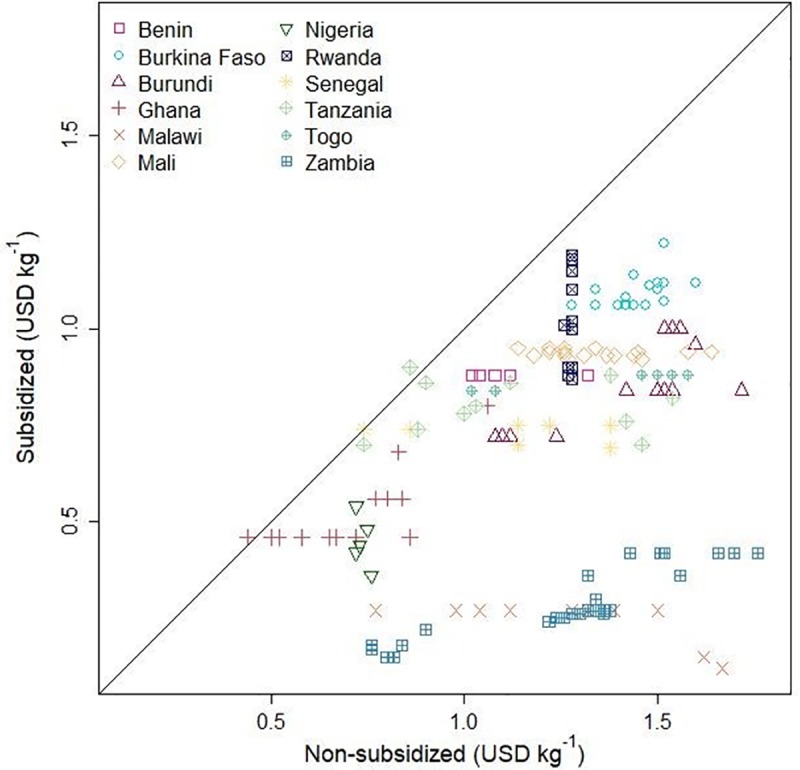
Subsidized urea price (USD kg^-1^) versus non-subsidized urea price (USD kg^-1^) for location where both urea prices were reported in each country.

We had 159 cases from twelve countries from Africa Fertilizer data where we had subsidized and non-subsidized fertilizer prices from the same town. In all the towns, subsidized prices were lower than non-subsidized prices. The slope of the regression models between subsidized and non-subsidized prices varied from 0.18 to 0.78 USD kg^-1^ with a mean of 0.61 ± 0.19 USD kg^-1^ (Fig 1).

#### Urea price relative to other types of fertilizer

**Fig 2:**
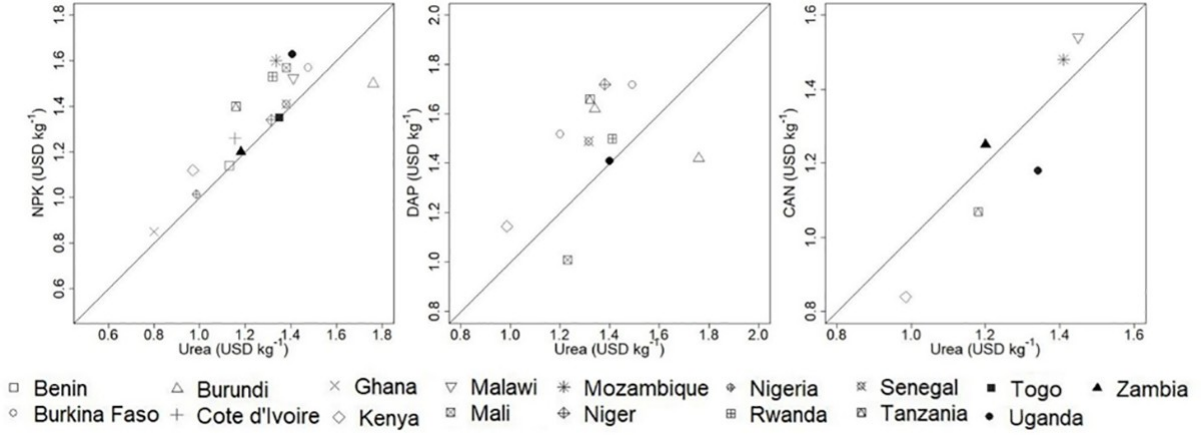
NPK median price (USD kg^-1^) versus urea median price (USD kg^-1^); DAP median price (USD kg^-1^) versus urea median price (USD kg^-1^); CAN median price (USD kg^-1^) versus urea median price (USD kg^-1^) for each country and location where both prices were reported. All the prices are non-subsidized. The black line represents *y = x*.

Ghana and Nigeria had the lowest median NPK prices (0.85 and 1.02 USD kg^-1^). The countries with the most expensive NPK were Burkina Faso, Burundi, Malawi, Mali, Mozambique, and Uganda (between 1.57 to 1.63 USD kg^-1^) (S1 Table). Except for Mozambique, these countries are landlocked. The lowest median DAP prices were in East African countries (Malawi and Kenya) and the highest median prices were in landlocked West African countries (Burkina Faso and Mali). CAN prices were only reported for East African countries. The lowest median prices were reported in Kenya and Tanzania and the highest were reported in Malawi and Mozambique (Fig 2 and S1 Table). Out of seventeen countries only in Burundi the median urea price was higher than the median NPK price; in Benin, Ghana, Niger, Nigeria, Senegal, Togo, and Zambia it was about the same, and in the other countries, the urea price was lower. Urea price was lower than DAP in most of the countries, but it was higher in Burundi and Malawi.

**Fig 3:**
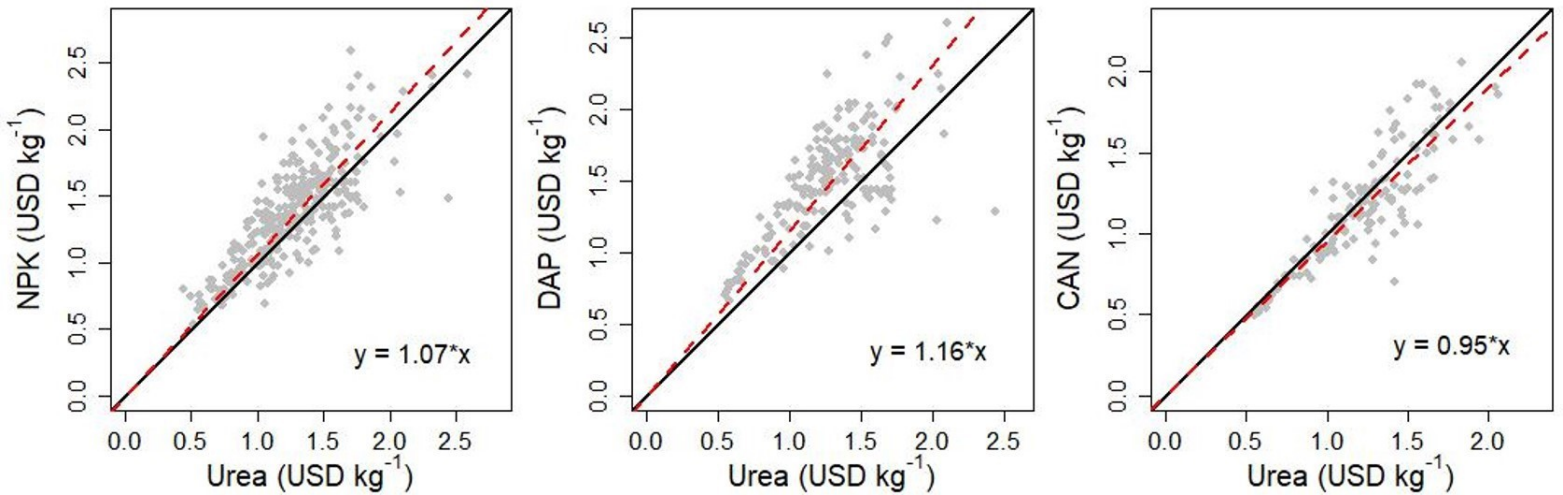
NPK price (USD kg^-1^) versus urea price (USD kg^-1^); DAP price (USD kg^-1^) versus urea price (USD kg^-1^); CAN price (USD kg^-1^) versus urea price (USD kg^-1^) for each location where both prices were reported. All the prices are non-subsidized. The black line represents *y = x*. The red line represents the fitted model.

The regression models for all locations showed a strong association between the price of urea and that of the other products. The slopes (that is, how much the price of NPK/DAP/CAN changes for a one dollar increase in the urea price) were 1.07 USD kg^-1^ for NPK; 1.16 USD kg^-1^ for DAP; and 0.95 USD kg^-1^ for CAN (Fig 3). For the NPK models fitted for each country, the average slope was 1.07 USD kg^-1^ (range: 0.85–1.18 USD kg^-1^) (S2 Table and S1 Fig). For the DAP models by country, the average slope was 1.13 USD kg^-1^ (range: 0.87–1.27 USD kg^-1^) (S3 Table and S2 Fig). For the CAN models by country, the average slope was 0.96 (range: 0.89–1.01 USD kg^-1^) (S4 Table and S3 Fig).

### Subnational urea price variation

#### Data quality

**Fig 4:**
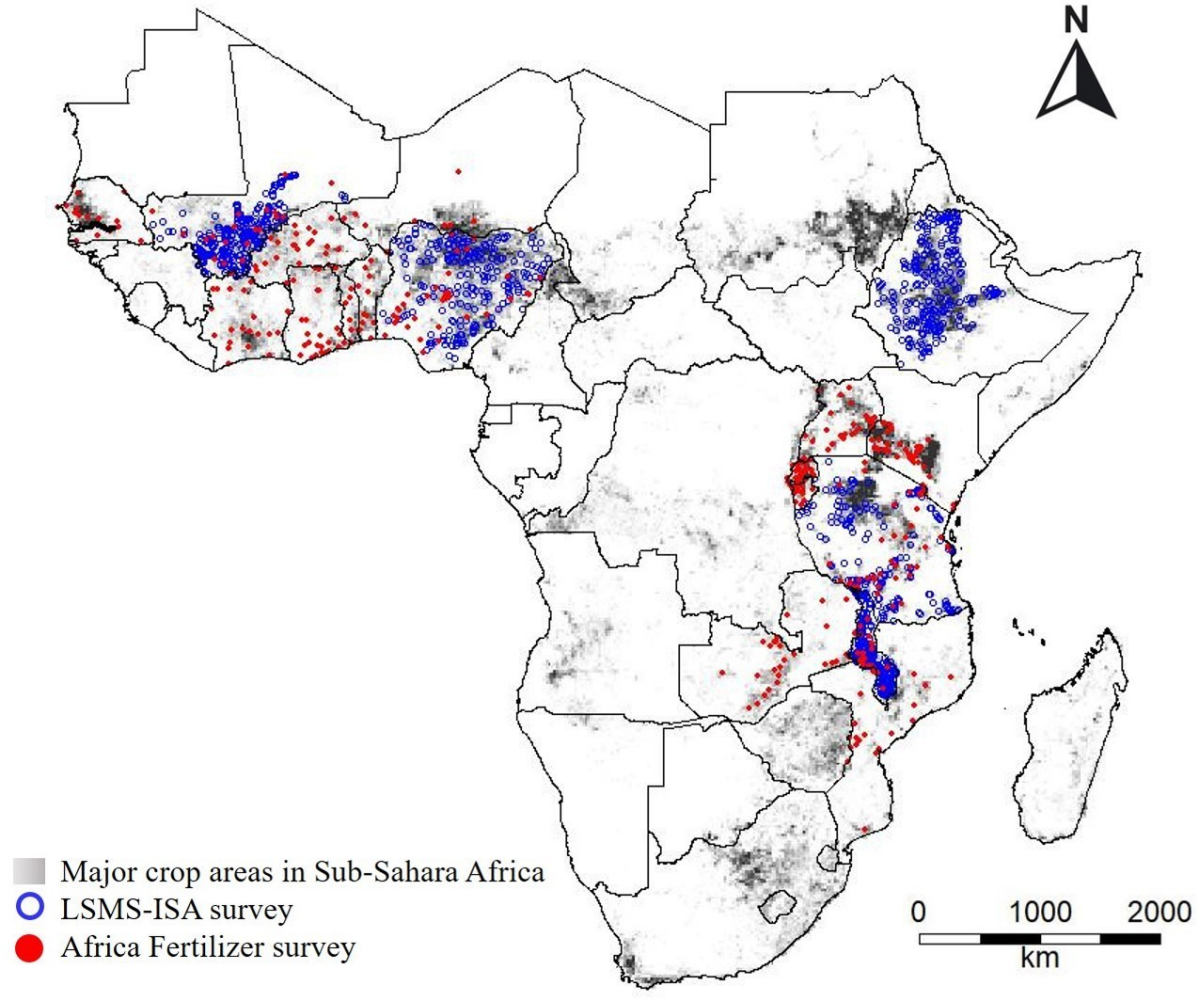
Major cropland area in Sub-Saharan Africa (gray areas), distribution of the urea price data from Africa Fertilizer (red dots) and the Living Standards Measurement Survey-Integrated Surveys on Agriculture (LSMS; blue dots), and country boundaries (black lines).

The density of the reported fertilizer prices varied between countries, but generally appeared to be a reasonably good spatial sample of the distribution of crop land in the analyzed countries (Fig 4). That is, price data was collected in areas with crop production, and excluded, for example, desert regions in the Sahel and Kenya, and the extensive protected areas in Tanzania. Nevertheless, some regions within countries had poor coverage. For example, observations in Zambia are concentrated in Central province, around Lusaka, the capital, and along the main north-south road that links Lusaka with major cities in Southern and Northern provinces, and there is only a single observation in western Zambia.

#### National level models

The cross-validation correlation coefficient of the national level ensemble models was between 0.17 and 0.83, with a mean of 0.52 while the E_I_ (RMSE) varied from 0.005 to 0.188, with a mean value of 0.095. The error relative to the Null model of no spatial variation, E_R_, was above 0.25 for ten countries. For Nigeria, the E_R_ was similar to the Null model and for Rwanda the E_R_ was negative, meaning that the national average was a better predictor of local prices than the spatially interpolated prices. The correlation coefficient of the models fitted with LSMS data varied between 0.08 to 0.42 with a mean equal to 0.27. The model built with LSMS data performed poorer than the model built with Africa Fertilizer data for Mali and Malawi (correlation coefficient for Mali: 0.08 vs 0.59 and Malawi: 0.28 vs 0.17 and E_R_ for Mali: -0.03 vs 0.28 and Malawi: 0.02 vs 0.28). The LSMS models for Nigeria was slightly better than the model fitted with Africa Fertilizer data, but still very low (correlation coefficient 0.32 vs 030 and E_R_: 0.02 vs -0.08). For Tanzania, the performance of the models was better for Africa Fertilizer but in this case LSMS also performed well (correlation coefficient 0.42 vs 0.58 and E_R_ 0.21 vs 0.26). For the regional models, the correlation between observed and predicted prices was 0.59 for East Africa and 0.54 West Africa and the models performed 21% and 16% better than the Null model (Table 3).

**Table 3:** Summary results for country level and regional models to predict the spatial price index (price divided by the national average) for urea and cross-validation results: Pearson’s correlation coefficient between observed and predicted data price. E_I_: Root mean square error (USD kg^-1^). E_0_: Mean square error of the Null model. E_R_: Relative mean square error, calculated as: 1-(E_I_ / E_0_). E_I_ and E_0_ are expressed for the relative index and for the relative index multiplied by the national average price (USD kg^-1^, values between parenthesis). For country-level models the data source was Africa Fertilizer, except where indicated otherwise.

Country-Data source	Median Price (USD kg^-1^)	Correlation coefficient	E_I_	E_0_	E_R_
Benin	1.12	0.52	0.136 (0.15)	0.172 (0.19)	0.21
Burundi	1.36	0.39	0.063 (0.09)	0.062 (0.08)	-0.02
Burkina Faso	1.42	0.22	0.048 (0.07)	0.056 (0.08)	0.14
Côte d’Ivoire	1.10	0.80	0.030 (0.03)	0.051 (0.06)	0.41
Ethiopia, LSMS	1.36	0.55	0.111 (0.15)	0.135 (0.18)	0.18
Ghana	0.73	0.79	0.097 (0.07)	0.118 (0.09)	0.19
Kenya	0.64	0.80	0.047 (0.03)	0.076 (0.05)	0.38
Malawi, Africa Fertilizer	0.76	0.17	0.067 (0.05)	0.093 (0.07)	0.28
Malawi, LSMS	1.29	0.28	0.359 (0.46)	0.367 (0.47)	0.02
Mali, Africa Fertilizer	1.28	0.59	0.075 (0.10)	0.104 (0.13)	0.28
Mali, LSMS	1.05	0.08	0.288 (0.30)	0.279 (0.29)	-0.03
Mozambique	1.20	0.63	0.108 (0.13)	0.145 (0.17)	0.26
Niger	1.34	0.39	0.048 (0.06)	0.062 (0.08)	0.23
Nigeria, Africa Fertilizer	0.74	0.32	0.057 (0.04)	0.053 (0.04)	-0.08
Nigeria, LSMS	0.80	0.30	0.185 (0.15)	0.188 (0.15)	0.02
Senegal	1.21	0.68	0.159 (0.19)	0.267 (0.32)	0.40
Rwanda	1.26	0.51	0.005 (0.01)	0.004 (0.01)	-0.25
Tanzania, Africa Fertilizer	0.97	0.58	0.148 (0.14)	0.200 (0.19)	0.26
Tanzania, LSMS	0.97	0.42	0.182 (0.18)	0.229 (0.22)	0.21
Tanzania, combined	0.97	0.52	0.188 (0.18)	0.224 (0.22)	0.12
Togo	1.48	0.45	0.040 (0.06)	0.059 (0.09)	0.32
Uganda	1.16	0.83	0.097 (0.11)	0.155 (0.18)	0.37
Zambia	1.24	0.50	0.042 (0.05)	0.049 (0.06)	0.14
East Africa*	1.20	0.59	0.101 (0.12)	0.128 (0.15)	0.21
West Africa*	1.02	0.54	0.120 (0.12)	0.142 (0.14)	0.16

*East Africa: Burundi, Ethiopia, Kenya, Mozambique, Malawi, Rwanda, Tanzania, Uganda, Zambia. West Africa: Benin, Burkina Faso, Côte d’Ivoire, Ghana, Mali, Niger, Nigeria, Senegal, Togo.

**Fig 5:**
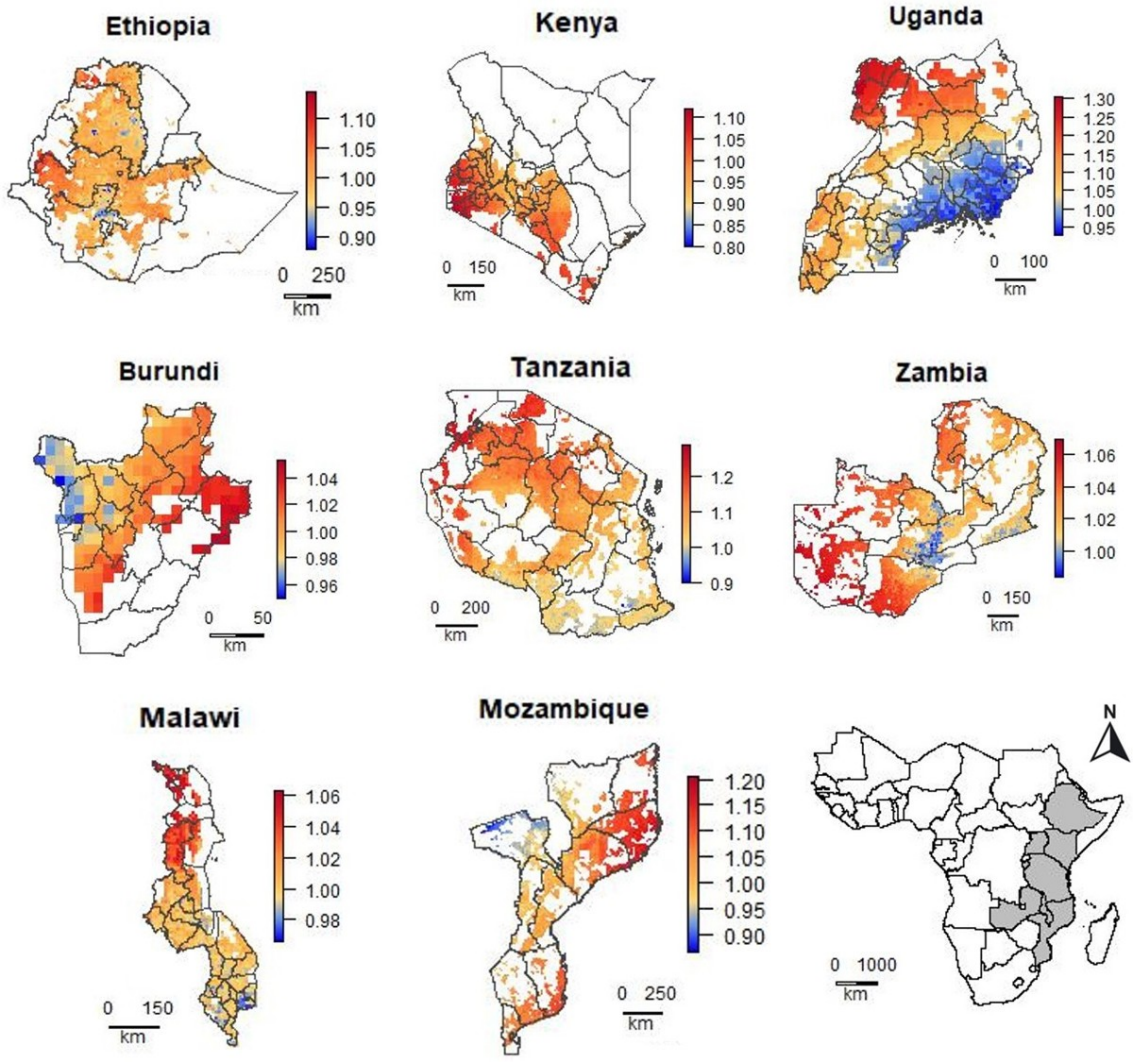
Predicted relative urea price of urea (local price divided by the observed median national price) for areas with crop land in eight East African countries. For Malawi, the prediction of the best-performing model is shown (Africa Fertilizer). For Tanzania, the prediction of the combined model (Africa fertilizer and LSMS) is shown.

**Fig 6:**
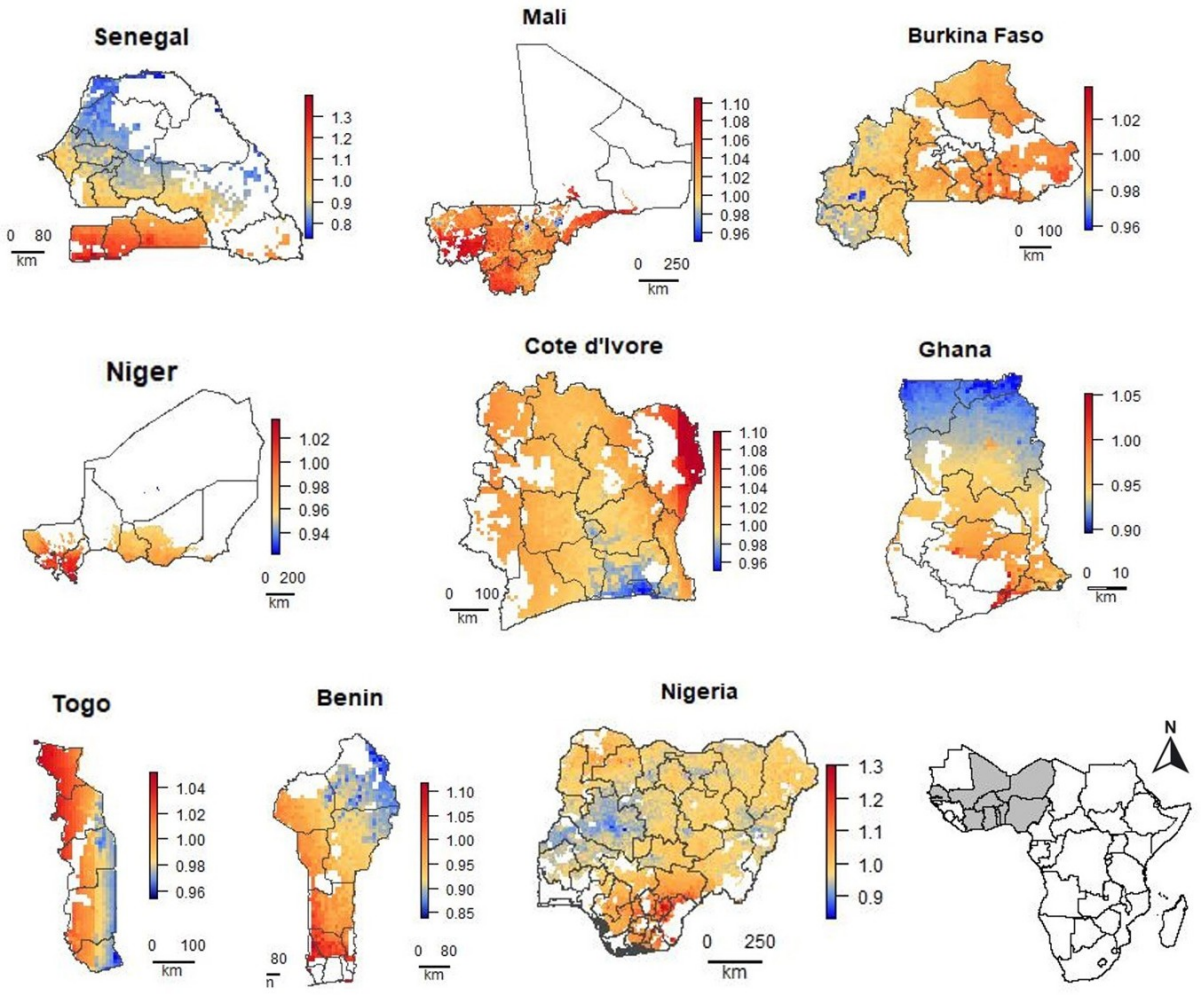
Predicted relative urea price (local price divided by the observed median national price) for areas with crop land in nine West African countries. For countries with two data sources (Africa Fertilizer and LSMS), the prediction of the best-performing model is shown.

The range in the predicted relative prices within the countries was between 0.59 and 1.40 (Figs 5 and 6). It was particularly high in Kenya (0.77–1.12), Nigeria (0.83–1.34), Senegal (0.73–1.40), Tanzania (0.90–1.29) and Uganda (0.93–1.30). It was low in Burkina Faso (0.96–1.04), Burundi (0.95–1.05), and Togo (0.94–1.05). For Rwanda, the spatial models performed worse than the Null model and the range in the relative prices was very low (0.99–1), so we assume no (systematic) variation in urea prices in these countries; and for that reason we did not include maps for Rwanda.

In several countries, for example, Côte d’Ivoire, Ethiopia, Kenya, Mali, Uganda, and Zambia, prices were lower closer to the capital and or other main markets. However, in Ghana and Benin the prices were lower in the north, further away from the capitals and main ports. In Tanzania, prices were relatively low in the south and coastal region and higher at the northwest. In Mozambique prices were lowest near the border with Zambia. In Senegal, prices are much higher south of The Gambia, which is a region of Senegal that is relatively difficult to reach from the other part of the country (Figs 5 and 6).

**Fig 7:**
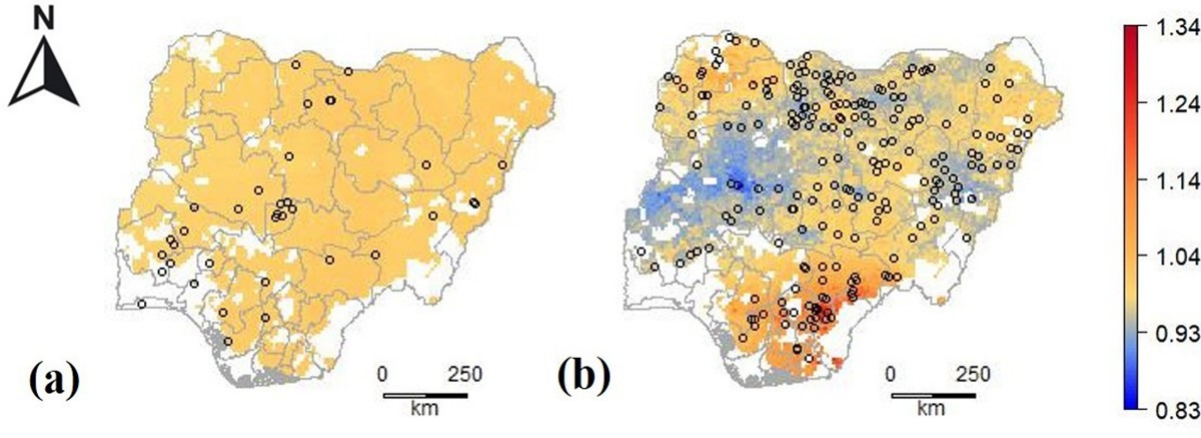
Predicted relative urea price (local price divided by the observed median national price) for areas with crop land in (a) Nigeria using Africa fertilizer data; (b) Nigeria using LSMS data.

For Nigeria the interpolation models were not much better than the Null model regardless the data source (E_R_: 0.08 and -0.02) (Table 3). The poor performance was associated with very limited variation in predicted prices for the Africa Fertilizer model, and for the LSMS model as well in most parts of the country. These results suggest that there is limited systematic spatial variation in fertilizer prices in Nigeria (Fig 7).

**Fig 8:**
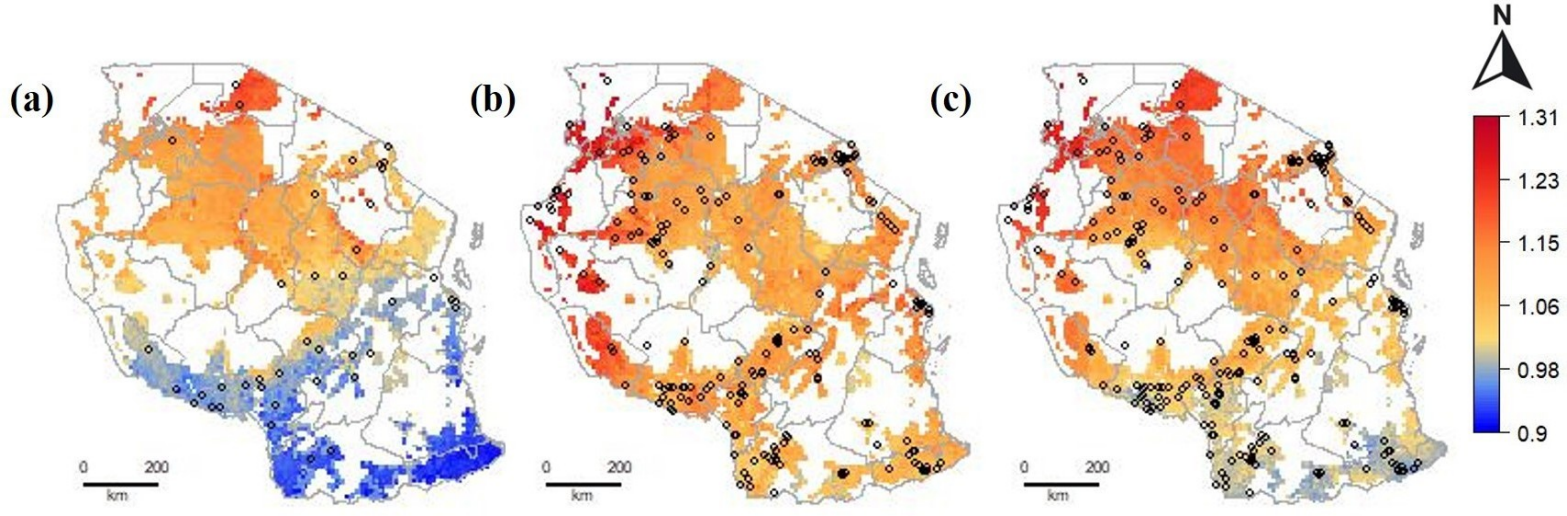
Predicted relative urea price (local price divided by the observed median national price) for areas with crop land in (a) Tanzania using Africa Fertilizer data; (b) Tanzania using LSMS data; (c) Tanzania using Africa Fertilizer data and a sub-sample of LSMS data with the sample size equal to Africa Fertilizer.

For Tanzania, models from both sources showed strong spatial variation. They both showed higher prices in the northwest, but they did not agree on prices in the south. Although the Africa fertilizer model performed slightly better than the LSMS model (E_R_: 0.26 vs 0.21); LSMS had much more data, and a better geographic coverage. There was no Africa Fertilizer data in the south where the predictions were most dissimilar. The performance of the model that combined Africa Fertilizer and LSMS data was not as good as that of the individual models (E_R_: 0.12) (Table 3). However, the spatial prediction of the combined model could be more realistic mostly in the south-east where no data from Africa Fertilizer was reported (Fig 8).

#### Regional models

**Fig 9:**
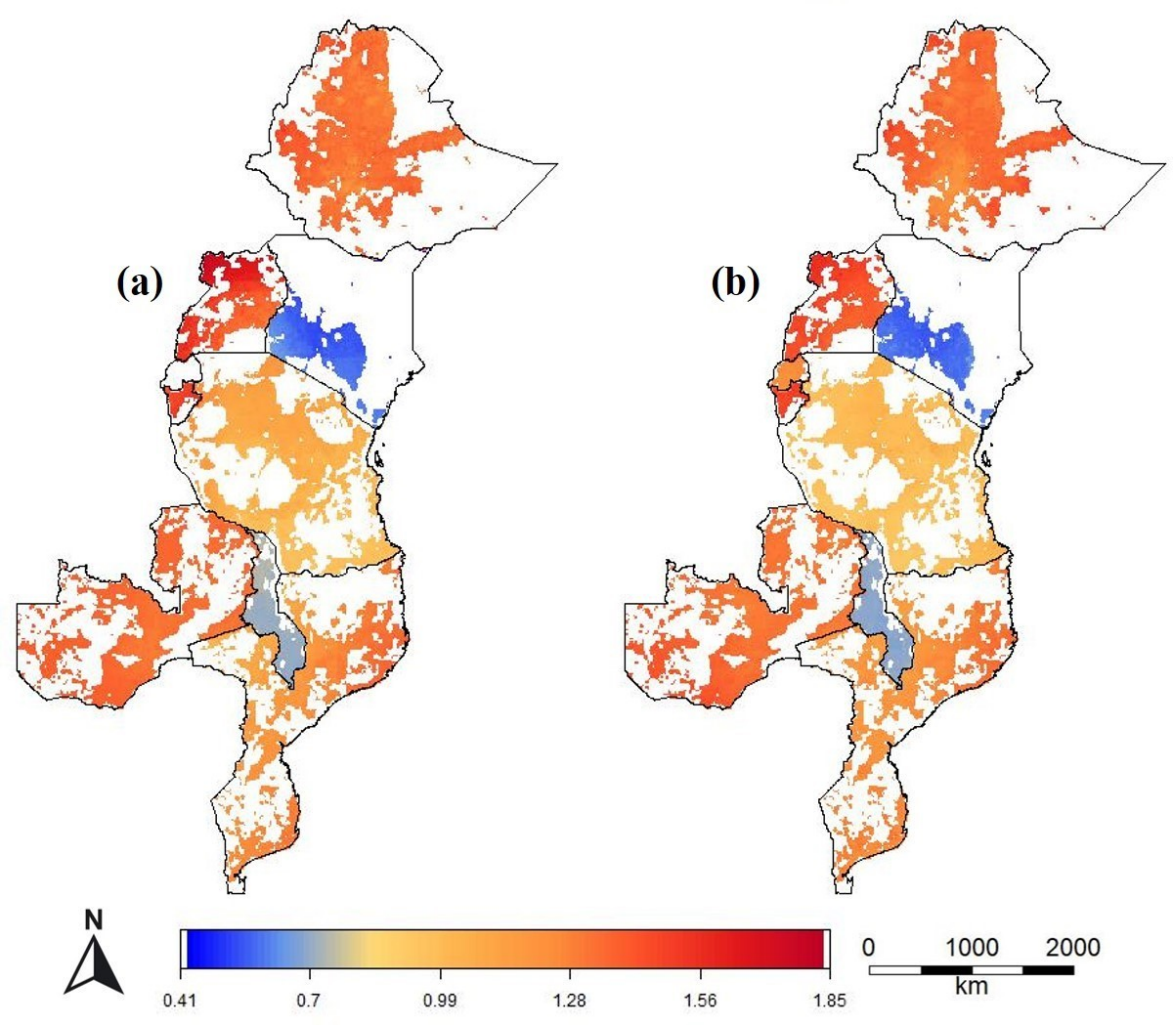
Urea price (USD kg^-1^) for crop land in East African countries predicted with (a) country level models; and (b) with a single regional model.

**Fig 10:**
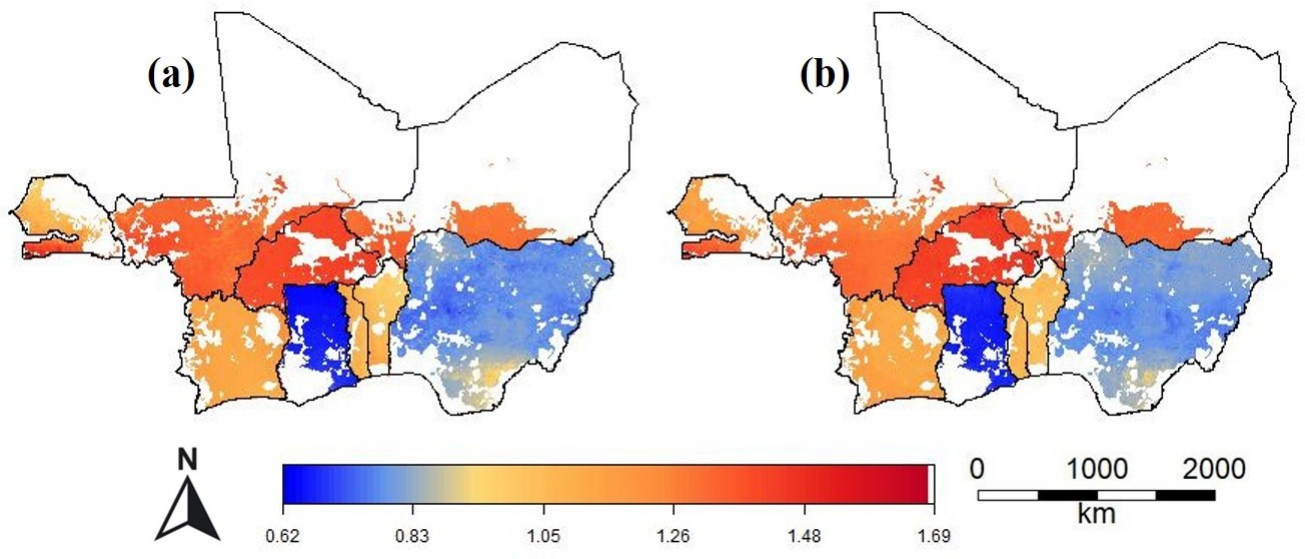
Urea price (USD kg^-1^) for crop land in West Africa predicted with (a) country level models and (b) with a single regional ensemble model.

The regional maps of the absolute prices show the strong effect of countries on fertilizer prices. Most of the variation is now between countries, instead of within countries. The predictions from a single regional model are similar to that of the combined country-based models, but with reduced spatial variation within countries (Figs 9 and 10).

#### Variable importance

**Fig 11:**
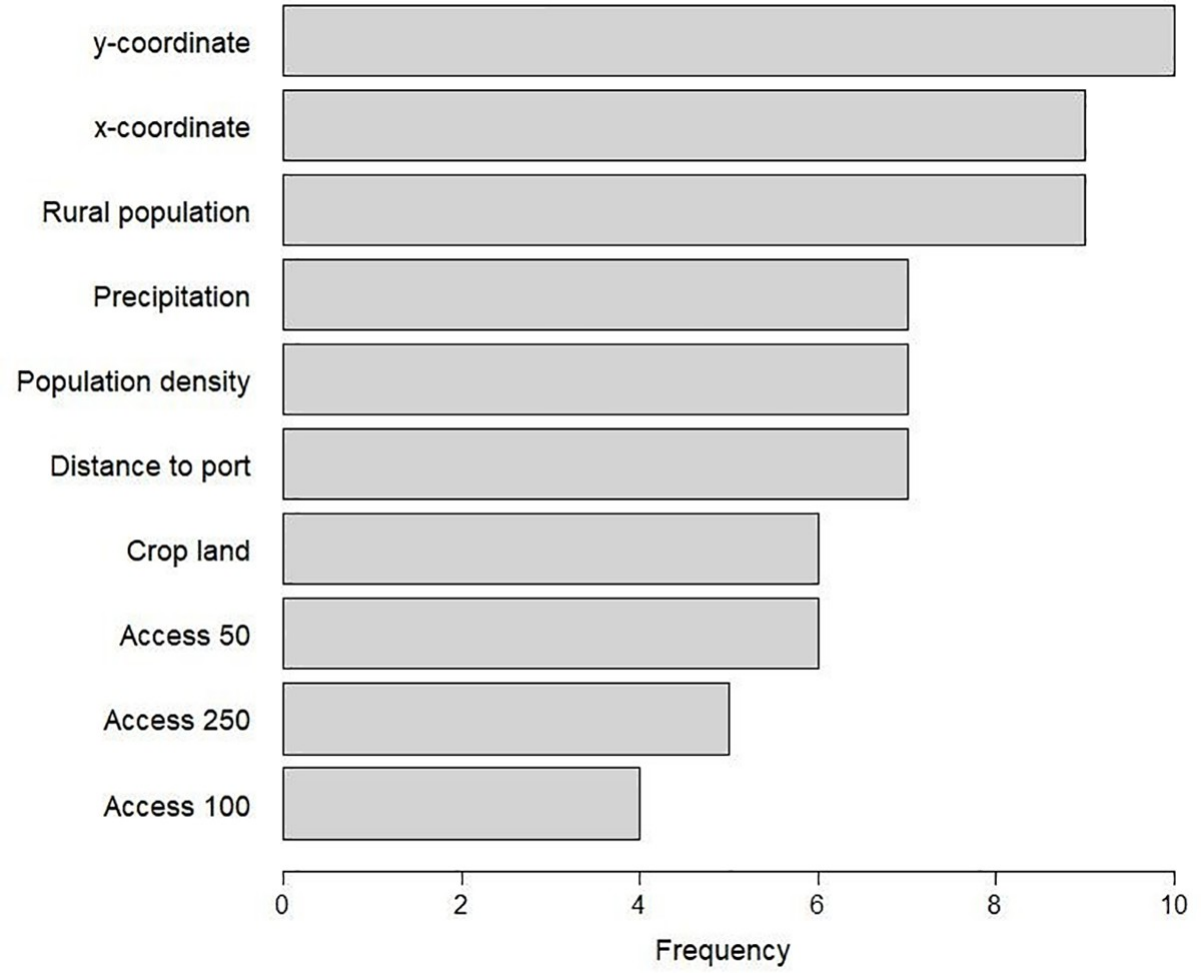
Variable importance across the eighteen-country level model. A variable is included if the % increase in Mean Square Error was more than 10% in a country-level Random Forest model. Access -50, -100, and -250: travel time (hours) to the nearest town with over 50,000 inhabitants, over 100,000 inhabitants, and over 250,000 inhabitants. Distance to port: travel time (hours) to the nearest port.

The most important variables in the country level Random Forest models were latitude and longitude, followed by rural population density, precipitation, and population density (Fig 11). For the East Africa regional model, the most important variables were y-coordinates (32%), cropland (32%), and rural population density (32% increase of MSE). For the West Africa regional model, the most important variables were longitude (28%), precipitation (21%), latitude, population density and distance to port (20% increase of MSE).

#### Transportation cost

LSMS reported where the farmers purchased the fertilizer (within or near the village, within or near the town, urban center, outside the district or region) for Ethiopia, Nigeria, and Tanzania. On average, 58% of the fertilizer was purchased within the village (70% in Ethiopia, 51% in Nigeria, and 52% in Tanzania); 22% near the village (18% in Ethiopia, 29% in Nigeria, and 18% in Tanzania); 15% within or near town (10% in Ethiopia, 18% in Nigeria, and 18% in Tanzania).

For Nigeria and Malawi, LSMS reported type of transportation and transportation costs farmers paid in order to obtain the fertilizer from the market. In Nigeria, for transportation 52% of the farmers used a motorcycle, 36% used a truck/bus/minibus, 5% a commercial bicycle, and the rest used either a wheelbarrow, own bicycle, animal cart, on foot, or a boat. Transportation cost range between 0.01 to 3.76 USD kg^-1^ of fertilizer, with a mean equal to 0.05 ± 0.17 USD kg^-1^. In this case, transportation cost (USD kg^-1^ of purchased fertilizer) by transportation type range between 0.01 to 0.05 USD kg^-^ (Table 4). On average the transportation cost was 4.35% of the total paid for the fertilizer price; for truck/bus/minibus this relationship was 1.93%, for wheelbarrow 2.13%, for own bicycle/animal cart/push cart 3.15%, for hired bicycle 3.68%, and for motorcycle 4.19%. The correlation between transportation cost and access to market (travel time in hours) over 250,000 inhabitants was 0.21. We fitted a linear regression with no-intercept between transportation cost and access to market and the slope (how much transportation cost changes, for a 1 hour increase in the travel time) was 0.59 USD. The average travel time to a market over 250,000 was 3.45 hours.

**Table 4:** Transportation type utilized by farmers to reach markets and purchase fertilizer, adoption rate (%), and median price paid (USD kg^-1^ of purchased fertilizer) reported by LSMS for Nigeria and Malawi.

Transportation type	Nigeria		Malawi	
	Adoption rate	Price	Adoption rate	Price
Bicycle taxi	5	0.03	15	0.04
Motorcycle	52	0.05	---	---
Own bicycle/Oxcart	---	---	29	0.06
On foot	---	---	28	---
Others	7	0.03	3	0.04
Truck/Bus/Minibus	36	0.01	25	0.08

In Malawi, for transportation 29% of the respondents used their own bicycle or oxcart, 28% went on foot, 25% used truck/bus/minibus; 15% bicycle taxi; and 3% others. Transportation cost ranged between 0.02 to 1.09 USD kg^-1^, with a mean equal to 0.06 ± 0.12 USD kg^-1^. In this case, transportation cost (USD kg^-1^ of purchased fertilizer) by transportation type varied between 0.04 to 0.08 USD kg^-1^ (Table 4). On average the transportation cost was 5.55% of the total paid for the fertilizer price. For truck/bus/minibus this relationship was 6.23%; for bicycle taxi was 5.11%; and for own bicycle/oxcart was 3.13%. Correlation between transportation cost and travel time was equal to 0.26 for markets over 50,000 inhabitants and 0.20 for markets over 250,000 inhabitants. On average travel time to markets over 50,000 was 3.98 hours and the slope between transportation cost and travel time was 0.43 USD.

## Discussion and conclusions

We have compiled a large amount of retail fertilizer price data to understand spatial variation in urea prices in eighteen countries in SSA. Our data covered much of the SSA cropland area, but we did not have data for a few large countries including South Africa, Sudan, and Zimbabwe. We found a considerable amount of spatial variation in urea prices in many countries. In most countries our spatial models fit the data reasonably well and outperformed the Null model of no spatial variation. Countries where the model performed very well, such as Côte d’Ivoire, Kenya, Senegal, Tanzania, and Uganda had a relatively high range in observed prices. Countries where the spatial prediction model did not perform very well were relatively small (e.g. Rwanda), and thus one might not expect as much spatial price variation. In Rwanda and Malawi, the fertilizer subsidy program may also be lowering the variation and complicating the pattern of spatial price variation. For instance, in Rwanda the price for subsidized prices was the same in all locations and the number of observations was higher than for non-subsidized urea. Nigeria was the only large country where the spatial prediction model did not perform well. While the predictions based on the LSMS showed spatial variation; the model predictions were poor, perhaps due to low data quality. The model fitted with Africa Fertilizer data had also a poor performance, perhaps due to the sparse spatial coverage.

The LSMS would appear to be an attractive data source by virtue of the large number of records of prices paid by farmers. However, models fit with LSMS data generally performed poorly, possibly due to data quality issues which are not immediately apparent from examination of the values reported, and/or the fact that farmers in a given locality may purchase fertilizer from different market locations which are imperfectly identified in the data. For countries where Africa Fertilizer data was also available, models fit with these data generally did better (or both models were very poor). This suggests that the LSMS as a source of local price data is compromised by data quality issues. While we were able to remove outliers, we had no way of correcting error for prices that were within a reasonable range of values. This problem has also been reported by others [21, 44]. The high number of unrealistic prices (excluded in our study as outliers) and poor model fit, suggest the need for better quality control mechanisms in this survey.

The consistency of the Africa Fertilizer data was generally much better. However, it is worth noting that these price data are only collected in towns and may not reflect the likely higher prices in more isolated rural areas. Also, because of the relatively low sample size, some important agricultural regions within countries were not sampled, making the predictions for these regions highly uncertain. An expansion of the Africa Fertilizer observation network within countries, and to other countries currently not covered would be very helpful to more fully understand fertilizer price variation.

Using regional models (i.e. pooled observations from multiple countries within a region), we predicted prices for the whole region with a single model. Regional models are attractive as they could be used to predict price variation in countries for which we have limited local market price data, but we did not investigate that here.

It has been reported that fertilizer prices in SSA increase when moving away from main markets due to transportation costs from the port or blending facilities [45, 22, 23, 24]. For example, a price gradient was reported for Uganda with retail prices steadily rising when moving away from Kampala [30]. We found this pattern in a number of countries, including Côte d’Ivoire, Ethiopia, Kenya, Mali and Uganda. However, there were clear exceptions too. For example, in Nigeria, Ghana, and Benin, prices went down when moving away from the coast. Possible factors influencing this pattern could be that: market prices in areas with higher demand are lower [46, 47, 27]. Aggregate fertilizer use in northern Nigeria is higher than in the south because it has a larger cultivated area and produces high value crops [48, 49]. In addition, northern states have traditionally provided greater fertilizer subsidies [50].

In some cases, imports of fertilizer from neighboring countries with lower prices may affect prices. For example, imported fertilizer from Malawi could be reducing the price in south-west Tanzania. The allocation of subsidized fertilizer, and the degree to which this leak into the non-subsidized market could also influence prices [51]. Political influence can affect the allocation of subsidies, with politically well-connected villages receive more input compared to less connected villages [52, 53, 54] or to reward loyalty [55, 56, 57].

It is important to know the price at the farmgate which includes the costs of the fertilizer *per se* plus the cost of reaching the market to get the product. Some authors reported little effect of changes in the cost of shipping fertilizer from the distributor to local retailer [21,58]. However, like [59], they found that farmers were sensitive to the costs of reaching retailers. We only had local transportation cost data for Malawi and Nigeria; our results suggest that the additional costs were 2% and 5.8% of the total amount paid for the fertilizer. Our estimated prices can be adjusted when using them in computations for on-farm costs.

Many studies of technology profitability have assumed spatially-constant input and output prices (e.g., [26, 27]), which is at odds with rural economic reality. A few studies have utilized “local” prices calculated as sub-national mean prices (e.g. at district or province level) from survey data (e.g. [18, 30, 60]). The sizes of such sub-national units often vary widely, and the boundaries are somewhat arbitrary, and there may be strong unobserved price variation within such areas. Our interpolation method is very likely an improvement over such approaches, particularly in countries where available spatial covariates perform well as predictors. We used an ensemble model of Random Forest Regression and Thin Plate Spline methods. A downside of using such algorithmic methods is that there is no direct way to estimate uncertainty. However, elaborate Bayesian geostatistical approaches have been developed to estimate uncertainty in similar contexts (e.g., [61, 62]) and these methods could be applied to price data as well. This is particularly relevant to account for the large uncertainty of the predictions in areas far away from any observations. It is important to note that cross-validation results may be inflated for such regions because, on average, model skill should decrease with the distance from a location to locations with known prices (*cf*., [63]).

An important avenue for future empirical work would be to evaluate the extent to which the subnational price variation we have documented is a useful explanatory factor for observed variation in smallholder fertilizer use in Sub-Saharan Africa, after controlling for local agronomic responses and output prices. One way to do that may be to integrate input (and output) price predictions into spatial crop models, and then evaluate the degree to which modeled fertilizer use profitability predicts observed fertilizer use rates across different locations.

Farmers in remote rural settings generally face less favorable input-output price ratios than farmers in less remote settings [58, 64]. These differences can likely help explain differential patterns of agricultural input use. Yet the specific ways in which these spatial patterns play out is often obscured by insufficient data on local prices, and the patterns we have revealed did not always follow simple expectations (e.g., prices being higher in coastal Ghana). The evidence we have compiled in this paper suggests that, while investments in more comprehensive and spatially representative price data collection would be very useful, we may utilize spatial price prediction models to extend the value of existing data to better reflect local price variation through interpolation. Even if imperfect, such estimates are almost certainly more usefully reflective of farmers’ economic realities than assumptions of spatially constant prices within a given country, for all but the smallest countries. We propose that spatial price estimation methods such as the ones we employ here may serve for better approximating heterogeneous economic market landscapes, until such time as truly comprehensive local market price information systems become available.

## Supporting information

S1 Table Median price for non-subsidized NPK, DAP, and CAN price (USD kg^-1^) per country. (DOCX)

S2 Table Results of linear regression model for NPK and urea prices for each country where both fertilizer types were reported. Number of observations (*n*) and estimated slope coefficient.
(DOCX)

S3 Table Results of linear regression model for DAP and urea prices for each country where both fertilizer types were reported. Number of observations (*n*) and estimated slope coefficient.
(DOCX)

S4 Table Results of linear regression model for CAN and urea prices for each country where both fertilizer types were reported. Number of observations (*n*) and estimated slope coefficient.
(DOCX)

S1 Fig Non-subsidized NPK price (USD kg^-1^) versus non-subsidized urea price (USD kg^-1^) for location where both urea prices were reported in each country. (DOCX)

S2 Fig Non-subsidized DAP price (USD kg^-1^) versus non-subsidized urea price (USD kg^-1^) for location where both urea prices were reported in each country. (DOCX)

S3 Fig Non-subsidized CAN price (USD kg^-1^) versus non-subsidized urea price (USD kg^-1^) for location where both urea prices were reported in each country. (DOCX)
