# Supplementary material for: Spatial variation in fertilizer prices in Sub-Saharan Africa
Source: PLoS One. 2020 Jan 14;15(1):e0227764. doi: 10.1371/journal.pone.0227764 (PMC6959603; doi:10.1371/journal.pone.0227764)
Supplement: S1 Table — (DOCX) [file pone.0227764.s001.docx]

**S1 Table.** Median price for non-subsidized NPK, DAP, and CAN price (USD kg^-1^) per country

| **Country** | **NPK** | **DAP** | **CAN** |
| --- | --- | --- | --- |
| Benin | 1.14 | --- | --- |
| Burkina Faso | 1.57 | 1.72 | --- |
| Burundi | 1.50 | 1.42 | --- |
| Côte d’Ivoire | 1.26 | --- | --- |
| Ghana | 0.85 | --- | --- |
| Kenya | 1.12 | 1.15 | 0.84 |
| Malawi | 1.53 | 1.01 | 1.54 |
| Mali | 1.57 | 1.72 | --- |
| Mozambique | 1.60 | 1.50 | 1.48 |
| Niger | 1.34 | 1.49 | --- |
| Nigeria | 1.02 | --- | --- |
| Rwanda | 1.53 | 1.66 | --- |
| Senegal | 1.41 | 1.41 | --- |
| Tanzania | 1.40 | 1.52 | 1.07 |
| Togo | 1.35 | --- | --- |
| Uganda | 1.63 | 1.62 | 1.18 |
| Zambia | 1.20 | --- | 1.25 |
