# Supplementary material for: Spatial variation in fertilizer prices in Sub-Saharan Africa
Source: PLoS One. 2020 Jan 14;15(1):e0227764. doi: 10.1371/journal.pone.0227764 (PMC6959603; doi:10.1371/journal.pone.0227764)
Supplement: S2 Table — Number of observations (n) and estimated slope coefficient. (DOCX) [file pone.0227764.s002.docx]

**S2 Table.** Results of linear regression model for NPK and urea prices for each country where both fertilizer types were reported. Number of observations (*n*) and estimated slope coefficient.

| **Country** | ***n*** | **Slope** |
| --- | --- | --- |
| Benin | 11 | 0.94 |
| Burkina Faso | 24 | 1.07 |
| Burundi | 19 | 0.85 |
| Côte d’Ivoire | 20 | 1.06 |
| Ghana | 28 | 1.03 |
| Kenya | 33 | 1.15 |
| Malawi | 20 | 1.05 |
| Mali | 19 | 1.14 |
| Mozambique | 24 | 1.15 |
| Niger | 10 | 1.03 |
| Nigeria | 36 | 1.01 |
| Rwanda | 21 | 1.18 |
| Senegal | 19 | 1.07 |
| Tanzania | 33 | 1.24 |
| Togo | 7 | 0.95 |
| Uganda | 24 | 1.18 |
| Zambia | 35 | 1.06 |
