# Supplementary material for: Spatial variation in fertilizer prices in Sub-Saharan Africa
Source: PLoS One. 2020 Jan 14;15(1):e0227764. doi: 10.1371/journal.pone.0227764 (PMC6959603; doi:10.1371/journal.pone.0227764)
Supplement: S3 Table — Number of observations (n) and estimated slope coefficient. (DOCX) [file pone.0227764.s003.docx]

**S3 Table.** Results of linear regression model for DAP and urea prices for each country where both fertilizer types were reported. Number of observations (*n*) and estimated slope coefficient.

| **Country** | ***n*** | **Slope** |
| --- | --- | --- |
| Burkina Faso | 11 | 1.16 |
| Burundi | 22 | 0.87 |
| Kenya | 46 | 1.27 |
| Mali | 19 | 1.22 |
| Niger | 6 | 1.07 |
| Rwanda | 22 | 1.26 |
| Senegal | 9 | 0.89 |
| Tanzania | 41 | 1.24 |
| Uganda | 23 | 1.20 |
