# Supplementary material for: Spatial variation in fertilizer prices in Sub-Saharan Africa
Source: PLoS One. 2020 Jan 14;15(1):e0227764. doi: 10.1371/journal.pone.0227764 (PMC6959603; doi:10.1371/journal.pone.0227764)
Supplement: S4 Table — Number of observations (n) and estimated slope coefficient. (DOCX) [file pone.0227764.s004.docx]

**S4 Table.** Results of linear regression model for CAN and urea prices for each country where both fertilizer types were reported. Number of observations (*n*) and estimated slope coefficient.

| **Country** | **n** | **Slope** |
| --- | --- | --- |
| Kenya | 46 | 0.89 |
| Malawi | 24 | 1.00 |
| Mozambique | 7 | 1.00 |
| Tanzania | 42 | 0.94 |
| Uganda | 21 | 0.94 |
| Zambia | 7 | 1.01 |
