# Supplementary material for: Spatial variation in fertilizer prices in Sub-Saharan Africa
Source: PLoS One. 2020 Jan 14;15(1):e0227764. doi: 10.1371/journal.pone.0227764 (PMC6959603; doi:10.1371/journal.pone.0227764)
Supplement: S1 Fig — (DOCX) [file pone.0227764.s005.docx]

**
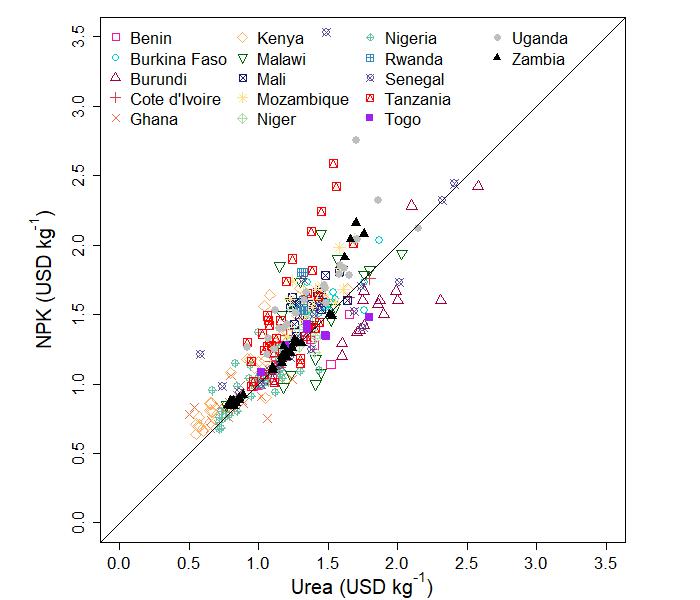
**

**S1 Fig.** Non-subsidized NPK price (USD kg^-1^) versus non-subsidized urea price (USD kg^-1^) for location where both urea prices were reported in each country.
